# LRRK2 kinase activity regulates synaptic vesicle trafficking and neurotransmitter release through modulation of LRRK2 macro-molecular complex

**DOI:** 10.3389/fnmol.2014.00049

**Published:** 2014-05-27

**Authors:** Maria D. Cirnaru, Antonella Marte, Elisa Belluzzi, Isabella Russo, Martina Gabrielli, Francesco Longo, Ludovico Arcuri, Luca Murru, Luigi Bubacco, Michela Matteoli, Ernesto Fedele, Carlo Sala, Maria Passafaro, Michele Morari, Elisa Greggio, Franco Onofri, Giovanni Piccoli

**Affiliations:** ^1^Division of Neuroscience, San Raffaele Scientific Institute and Vita-Salute UniversityMilan, Italy; ^2^Department of Molecular and Cellular Pharmacology, National Research Council, Neuroscience InstituteMilan, Italy; ^3^Department of Experimental Medicine, University of GenovaGenova, Italy; ^4^Department of Biology, University of PadovaPadova, Italy; ^5^Department of Medical Biotechnology and Translational Medicine, University of MilanMilan, Italy; ^6^Department of Medical Science and National Institute of Neuroscience, University of FerraraFerrara, Italy; ^7^Humanitas Clinical and Research Center, Pharmacology and Brain PathologyRozzano, Italy; ^8^Department of Pharmacy, University of GenoaGenoa, Italy

**Keywords:** LRRK2, kinase, presynaptic vesicle, synaptic activity, protein interaction

## Abstract

Mutations in Leucine-rich repeat kinase 2 gene (*LRRK2*) are associated with familial and sporadic Parkinson's disease (PD). LRRK2 is a complex protein that consists of multiple domains executing several functions, including GTP hydrolysis, kinase activity, and protein binding. Robust evidence suggests that LRRK2 acts at the synaptic site as a molecular hub connecting synaptic vesicles to cytoskeletal elements *via* a complex panel of protein-protein interactions. Here we investigated the impact of pharmacological inhibition of LRRK2 kinase activity on synaptic function. Acute treatment with LRRK2 inhibitors reduced the frequency of spontaneous currents, the rate of synaptic vesicle trafficking and the release of neurotransmitter from isolated synaptosomes. The investigation of complementary models lacking LRRK2 expression allowed us to exclude potential off-side effects of kinase inhibitors on synaptic functions. Next we studied whether kinase inhibition affects LRRK2 heterologous interactions. We found that the binding among LRRK2, presynaptic proteins and synaptic vesicles is affected by kinase inhibition. Our results suggest that LRRK2 kinase activity influences synaptic vesicle release *via* modulation of LRRK2 macro-molecular complex.

## Introduction

Parkinson's disease (PD) is an age-related neurodegenerative disease affecting 2% of the population above 65-years and is clinically characterized by bradykinesia, rigidity, and resting tremor. The neuropathological hallmark of the disease is the progressive loss of dopaminergic neurons in the *substantia nigra* (Moore et al., 2005; Hardy et al., 2006). Although the majority of cases are idiopathic, mutations in the Leucine-rich repeat kinase 2 (LRRK2) gene (PARK8; OMIM 609007) cause late-onset PD. LRRK2 mutations account for up to 13% of familial PD cases compatible with dominant inheritance (Paisan-Ruiz et al., 2004; Zimprich et al., 2004) and have been identified in 1–2% of sporadic PD patients (Aasly et al., 2005; Berg et al., 2005). LRRK2 is a large protein encompassing several functional domains including a kinase domain with feature similar to mitogen activated protein kinase kinase kinases (MAPKKK) and receptor-interacting protein kinases (RIPK) (Bosgraaf and Van Haastert, 2003; Guo et al., 2006). Several single nucleotide variants have been identified in LRRK2 (Brice, 2005). While only the common G2019S mutation, located in the kinase domain, has been consistently associated with increased kinase activity *in vitro* (West et al., 2005; Gloeckner et al., 2006; Greggio et al., 2006), a recent study monitoring LRRK2 autophosphorylation at Ser 1292 suggested that other pathogenic mutants possess augmented activity in the cellular context (Sheng et al., 2012). Up to now few LRRK2 substrates have been identified in *in vitro* studies, but none has been convincingly proved *in vivo*, leaving the pathophysiological relevance of the kinase activity unclear. Instead, several lines of evidence suggest that kinase activity is linked to LRRK2 dimerization (Greggio et al., 2008; Sen et al., 2009; Civiero et al., 2012) as well as subcellular distribution (Berger et al., 2010) and regulates binding to 14-3-3 proteins (Nichols et al., 2010). Accumulating data correlate LRRK2 to synaptic functions. Several studies suggested that LRRK2 is part of a protein complex that influences the trafficking of synaptic vesicles belonging to the recycling pool (Shin et al., 2008; Piccoli et al., 2011; Matta et al., 2012). The description of synaptic phenotype in LRRK2 mutant models (Tong et al., 2009; Migheli et al., 2013; Yun et al., 2013) further underlines the tight link among LRRK2, synaptic vesicle trafficking and neurotransmitter release. In this study we investigated the functional impact of LRRK2 kinase activity on presynaptic function and we determined functional properties of neurons upon LRRK2 pharmacological inhibition. A combination of electrophysiological, biochemical and imaging analyses suggested that LRRK2 inhibition impacts synaptic transmission acting on the organization of LRRK2 macro-molecular complex at the presynaptic site.

## Materials and methods

### Animals, neuron cultures, and drugs

Non-transgenic wild-type (WT) and LRRK2 knock-out (KO) mice, back-crossed on a C57BL/6J strain, were obtained from Mayo Clinic (Jacksonville, FL, USA) through a collaboration with Prof. Matthew Farrer and Dr. Heather Melrose (Hinkle et al., 2012). Animals were kept following guidelines of Ministry of Education, Universities and Research (MIUR). Neuron cultures were prepared from either mouse cortexes or hippocampi obtained from embryonic day 15.5–16.5 mice (C57BL/6J). High-density (750–1000 cells/mm^2^) and medium-density (150–200 cells/mm^2^) neuron cultures were plated and grown as described on 12-well plastic tissue culture plates (Iwaki; Bibby Sterilin Staffordshire, UK) or on 12 mm diameter coverslips put into 24-well plastic tissue culture plates (Iwaki) (Piccoli et al., 2007). IN-1 and GSK-2578215A compounds (Tocris Bioscience, Bristol, UK) or DMSO were added to culture media at the concentrations indicated through the text.

### Plasmids and protein purification

N-terminal 3xFLAG and myc hLRRK2 full length (hereinafter FLAG-LRRK2 and myc-LRRK2), N-terminal FLAG hLRRK2 A2106T (a kind gift of Prof. Dario Alessi, MRC, University of Dundee), LRRK2 silencing and control viral constructs vectors have been already described (Bauer et al., 2009; Nichols et al., 2009; Civiero et al., 2012). FLAG-LRRK2 was purified via affinity chromatography using FLAG-M2 agarose beads (Sigma Aldrich) as previously described (Civiero et al., 2012) from HEK293T cells transfected by lipofection using Lipofectamine 2000 (Life Technologies Carlsbad, CA, USA) according to manufacturer's instructions. Viral particles were produced as in (Bauer et al., 2009). Neurons were infected at DIV4 and processed when indicated.

### Immuno-precipitation and antibodies

Immunoprecipitation was performed as described previously (Onofri et al., 2007) using 25 μ l of settled prewashed protein G-Sepharose beads (GE-Healthcare, Freiburg, Germany) to precipitate the immunocomplexes. NaCl 150 mM, Tris 50 mM (pH 7.4), NP-40 (1% v/v), SDS (0.1% v/v) and protease and phosphatase inhibitors extracts of Percoll-purified synaptosomes obtained from rat cerebral cortex were incubated for 2 h at RT in absence or in presence of IN- 1 (1 μ M) with anti-LRRK2 antibodies (10 μg/sample; MJFF C41-2 Abcam, Cambridge UK) or a control rabbit IGg (10 μ g/sample; Sigma-Aldrich, St. Louis, MO, USA). The eluted proteins were separated by SDS-PAGE, transferred onto nitrocellulose membrane (GE-Healthcare) and analyzed by western-blotting. Antibodies list includes rabbit anti LRRK2 1:500 MJFF C41-2, rabbit anti LRRK2 P-Ser 935 UDD2 10(12) (Abcam), rabbit anti synapsin I 1:500 (Synaptic System, Goettingen, Germany), mouse anti actin 1:1000, mouse anti FLAG 1:1000, mouse anti myc 1:1000, mouse anti synaptophysin 1:1000 (Sigma-Aldrich St. Louis, MO, USA). The secondary antibodies (HRP-conjugated anti-mouse, anti-rabbit) (BIORAD, Hercules, CA, USA) were used in a ratio of 1:5000 coupled with the ECL chemiluminescence detection system. Immunoblots were quantified by densitometric analysis of the fluorograms (Quantity One software, Bio-Rad) obtained in the linear range of the emulsion response.

### *In vitro* kinase assay

GST-LRRK2^970−2527^ (Life technologies) at the concentration of 30 nM were incubated with 500 μM LRRKtide, 100 μM ^33^P-ATP (0.5 μCi) in kinase reaction buffer consisting of 25 mM Tris-HCl (pH7.5), 5 mM beta-glycerophosphate, 2 mM dithiothreitol (DTT), 0.1 mM Na_3_VO_4_, 10 mM MgCl_2_ and increasing concentrations of inhibitors at 30°C for 1 h. Reactions were carried out in triplicate and spotted onto P81 phosphocellulose. Following different washing of phosphocellulose membranes with 75 mM phosphoric acid, ^33^P incorporation into LRRKtide was quantified with Cyclone (Perkin Elmer, Alameda, CA, USA).

### Size exclusion chromatography

Cells transiently transfected with FLAG-LRRK2 wild-type were solubilized in lysis buffer containing 20 mM Tris-HCl pH 7.5, 150 mM NaCl, 1 mM EDTA, 1% Triton X-100, 2.5 mM sodium pyrophosphate, 1 mM beta-glycerophosphate, 1 mM NaVO_4_, protease inhibitor cocktail (Sigma-Aldrich) and lysates were cleared for 30 min at 14,000 xg. When appropriate, proteins were further purified via FLAG immunoprecipitation as described above. Cleared lysates (0.5 ml; 5 mg total proteins) or purified proteins (0.5 ml; 1.3 μg of purified protein) were injected and separated on a Superose 6 10/300 column (GE Healthcare). The column was preequilibrated with buffer (20 mM Tris-HCl pH 7.5, 150 mM NaCl and 0.07% Triton X-100) and used at a flow rate of 0.5 ml/min. Elution volumes of standards were 7.5 ml for Blue Dextran (V0), 11.5 ml for hemocyanin from Carcinus aestuarii (900 kDa), 12 ml for thyreoglobin (669 kDa), 14 ml for ferritin (440 kDa). When appropriate, inhibitors (1 μM IN-1 and 1 μM GSK-2578215A) were applied for 90 min before lysis and kept throughout the following purification steps, including equilibration of chromatographic mobile phase. Chromatographic fractions were analyzed by dot blot. One microliter of each fraction from SEC was applied onto a nitrocellulose membrane. The membrane was blocked with 10% (w/v) milk in TBS plus 0.1% Triton (TBS-T) for 1 h and subsequently incubated with mouse monoclonal anti-Flag M2-peroxidase (Sigma-Aldrich). Immunoreactive proteins were visualized using enhanced chemiluminescence plus (ECL plus, GE Healthcare).

### Synaptic vesicle purification and LRRK2 binding assays

Synaptic vesicles (SV) were obtained from rats by homogenization of the isolated forebrains and finally purified through the step of controlled-pore glass (CPG) chromatography (Huttner et al., 1983). After elution, purified SV were centrifuged for 2 h at 175,000 × *g* and resuspended at a protein concentration of 1–2 mg/ml in 0.3 M glycine, 5 mM HEPES, 0.02% sodium azide, pH 7.4 (glycine buffer). Protein concentrations were determined by the Bradford or BCA assays. SDS-PAGE was performed according to Laemmli (1970). For the dissociation of endogenously bound LRRK2 purified SV (40 μ g/sample) were incubated for 1 h at 30°C with or without IN-1 (1 μ M) in glycine buffer plus 30 mM NaCl, 25 mM Tris/HCl, 2 mM DTT, 10 mM MgCl_2_ protease and phosphatase inhibitors. After the incubation, LRRK2 bound to SV were separated by soluble LRRK2 by high-speed centrifugation (400,000 × g for 45 min) (Messa et al., 2010). Aliquots of the resuspended pellets were subjected to SDS–PAGE and subsequent Western blotting with anti LRRK2 MJFF C41-2 (Abcam) antibody. The recovery of SV, used to correct the amounts of LRRK2 bound to SV, was determined by Western blotting with anti-synaptophysin antibody (kind gift of Prof. Paul Greengard The Rockefeller University New York USA). The binding of purified FLAG-LRRK2 to native SV was performed like below. SV (10 μ g/sample) were incubated for 1 h at 0°C with FLAG-LRRK2 (50 nM) in glycine buffer plus 30 mM NaCl, 25 mM Tris/HCl, 2 mM DTT, 10 mM MgCl2 protease and phosphatase inhibitors and 1.0 μg/ml bovine serum albumin in absence or in presence of IN-1 (1 μM). After incubation, SV-bound FLAG-LRRK2 was separated by high-speed centrifugation (400,000 g for 45 min). Aliquots of the resuspended pellets were subjected to immunoblotting with anti-FLAG (Sigma-Aldrich) antibodies. The recovery of SV was determinated like above.

### Exo-endocytotic assay

The endocytosis assay to monitor SV recycling was performed using rabbit polyclonal antibodies directed against the intravesicular domain of synaptotagmin1 (Synaptic System), applied for 5 min if not indicated otherwise at RT on the cultures, as described previously (Matteoli et al., 1992). Incubations with the antibody (1:400) were performed in Tyrode solution containing 124 mM NaCl, 5 mM KCl, 2 mM MgCl_2_, 30 mM glucose, 25 mM HEPES, pH 7.4 and 2 mM CaCl_2_. After fixation and permeabilization, a synaptophysin counter staining with mouse anti synaptophysin, 1:400 (Sigma-Aldrich) visualized the totality of SV. Acquired images were processed and quantitatively analyzed with ImageJ software as previously described (Verderio et al., 1999). Briefly, GFP positive processes were manually tracked and the number of synaptotagmin and synaptophysin positive clusters and synaptophysin positive clusters present in the region of interest were automatically counted.

### Neurotransmitter release

Synaptosome were isolated from cerebral cortex (fronto-temporal areas) as described previously (Marti et al., 2003; Mela et al., 2004). The synaptosomal pellet was resuspended in oxygenated (95% O2, 5% CO2) Krebs solution (mM: NaCl 118.5, KCl 4.7, CaCl2 1.2, MgSO4 1.2, KH2PO4 1.2, NaHCO3 25, glucose 10). One millilitre aliquot of the suspension (~0.35 mg protein) was slowly injected into nylon syringe filters (outer diameter 13 mm, 0.45 μM pore size, internal volume of about 100 μl; Teknokroma, Barcelona, Spain) connected to a peristaltic pump. Filters were maintained at 36.5°C in a thermostatic bath and superfused at a flow rate of 0.4 ml/min with a preoxygenated Krebs solution. Under the superfusion conditions adopted in the present study, the fast and continuous removal of endogenous substances released by nerve terminals rules out that endogenous glutamate is uptaken by glutamate transporters, or even activates autoreceptors. Sample collection (every 3 min) was initiated after a 20 min period of filter washout. The effect of IN-1 was evaluated on both spontaneous efflux and K+-stimulated neurotransmitter outflow. IN-1 (3 μM) was added to the perfusion medium 9 min before a 90 s pulse of 15 mM KCl, and maintained until the end of the experiment. In other experiments purified synaptosomes were prepared on Percoll gradients (Sigma-Aldrich) and incubated at 37°C for 15 min in presence of 0.03 μM [^3^H]D-aspartate (Marte et al., 2010). A 90 s period of depolarization was applied at *t* = 39 min of superfusion with 15 mM KCl, substituting for an equimolar concentration of NaCl. IN-1 1 μM was added 9 min before depolarization. Fractions collected and superfused synaptosomes were counted for radioactivity by liquid scintillation counting. The efflux of radioactivity in each fraction has been expressed as a percentage of the total radioactivity present in synaptosomes at the onset of the fraction collected (fractional rate). Depolarization-evoked neurotransmitter overflow was calculated by subtracting the transmitter content of the two 3-min fractions, representing the basal release, from that in the two 3-min fractions collected during and after the depolarization pulse.

### Slice electrophysiology

C57Bl/6J mice were anesthetized in a chamber saturated with chloroform and then decapitated. The brain was rapidly removed and placed in an ice-cold solution containing 220 mM sucrose, 2 mM KCl, 1.3 mM NaH_2_PO_4_, 12 mM MgSO_4_, 0.2 mM CaCl_2_, 10 mM glucose, 2.6 mM NaHCO_3_ (pH 7.3, equilibrated with 95% O2 and 5% CO2). Coronal hippocampal slices (thickness, 250–300 μm) were prepared with a vibratome VT1000 S (Leica, Wetzlar Germany) and then incubated first for 40 min at 36°C and then for 30 min at room temperature in artificial CSF (aCSF), consisting of (in mM) 125 NaCl, 2.5 KCl, 1.25 NaH_2_PO_4_, 1 mM MgCl_2_, 2 mM CaCl_2_, 25 mM glucose, and 26 mM NaHCO_3_ (pH 7.3, equilibrated with 95% O_2_ and 5% CO_2_). Slices were then divided into 2 experimental groups: the first one was the control group and the second one was the group of slices incubated with the inhibitor 1 at concentration of 2 μM for at least 2 h. Slices were transferred to a recording chamber perfused with aCSF, where the concentration of CaCl_2_ was increased to 4 mM and MgCl2 decreased to 0.5 mM, due to the low frequency of miniature excitatory post-synaptic currents (mEPSCs) in CA1 hippocampus, at a rate of ~ 2 ml/min and at 38°C. Whole-cell patch-clamp electrophysiological recordings were performed with an Axon Multiclamp 700 B amplifier (Molecular devices, Sunnyvale, CA USA) and using an infrared-differential interference contrast microscope. Patch microelectrodes (borosilicate capillaries with a filament and an outer diameter of 1.5 μm; Sutter Instruments, Novato, CA USA) were prepared with a four-step horizontal puller (Sutter Instruments) and had a resistance of 3–5 MΩ.

mEPSCs were recorded at a holding potential of −65 mV with an internal solution containing: 126 mM K-gluconate, 4 mM NaCl, 1 mM EGTA, 1 mM MgSO_4_, 0.5 mM CaCl_2_, 3 mM ATP (magnesium salt), 0.1 mM GTP mM (sodium salt), 10 mM glucose, 10 mM HEPES (pH adjusted to 7.3 with KOH). Access resistance was between 10 and 20 MΩ; if it changed by >20% during the recording, the recording was discarded. All glutamatergic currents were recorded in the presence of bicuculline (20 μM) in the external solution, to block the GABAergic transmission, and lidocaine (500 μM), to block the action potentials onset. Currents through the patch-clamp amplifier were filtered at 2 kHz and digitized at 20 kHz using Clampex 10.1 Software (Molecular Devices). Analysis was performed offline with Clampfit 10.1 software (Molecular Devices).

### Electrophysiological recordings of cultured neurons

Whole-cell voltage clamp recordings were performed using a MultiClamp 700 A amplifier (Molecular devices) coupled to a pCLAMP 10 Software (Molecular Devices), and using an inverted Axiovert 200 microscope (Zeiss, Oberkochen Germany). Patch electrodes, fabricated from thick borosilicate glasses (Sutter Instruments) were pulled and fire-polished to a final resistance of 3–5 MΩ using a two-stage puller (Narishige, Japan). Experiments were performed at room temperature (20–25°C) in the external control solution KRH (125 mM NaCl, 5 mM KCl, 1.2 mM MgSO_4_, 1.2 mM KH_2_PO, 2 mM CaCl_2_, 6 mM D-glucose, and 25 mM HEPES/NaOH, pH 7.4). The age of the patched neurons ranged between 13 and 16 DIV. Recordings were performed keeping neurons at holding potential of -70 mV, in the presence of 1 μM TTX and using the following internal solution (Potassium Gluconate—KGluc): 130 mM KGluc, 10 mM KCl, 1 mM EGTA, 10 mM HEPES, 2 mM MgCl_2_, 4 mM MgATP, 0.3 mM Tris-GTP (pH 7.4, adjusted with KOH). Traces were acquired at 10 kHz and lowpass filtered at 4 kHz. Recordings with either leak currents >300 pA or series resistance >20 MΩ were discarded. Series resistance was monitored during experiments and recordings with changes over 20% control during experiments were also discarded. mEPSC traces were analyzed using MiniAnalysis Program (Synaptosoft Decatur, GA USA) with a threshold of 10 pA. Only events exceeding the baseline noise by >2 SDs were considered. The mean mEPSC frequency for CTRL neurons was 1.23646 ± 0.13746 Hz (mean ± *SE*).

### Statistical analysis

All data are expressed as mean ± standard error of the mean (SE). Data were analyzed with an unpaired Student's *t*-test (two groups) or ANOVA followed by Dunn's *post-hoc* test (more than two groups). The indication of number of experiment (n) and level of significance (p) are indicated throughout the text.

## Results

### LRRK2 kinase inhibition impairs synaptic transmission

**Figure 1:**
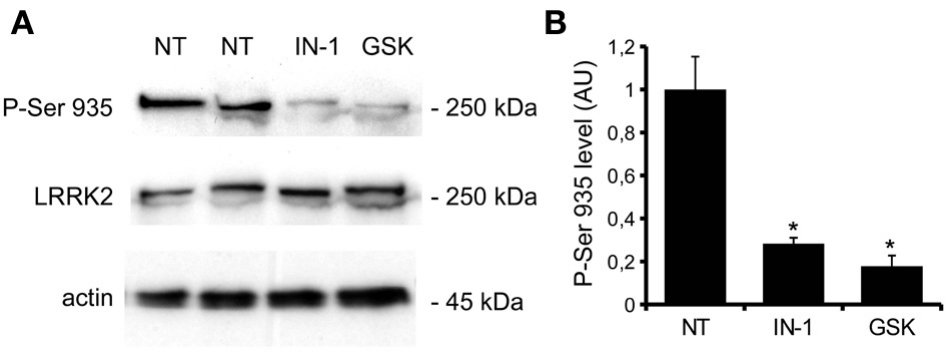
**IN-1 and GSK inhibit LRRK2 activity *ex-vivo*. (A)** Acute hippocampal slices were incubated in regular aCSF (NT) or in aCSF + IN-1 (IN-1) or GSK (GSK) both 1 μ M for 2 h at RT. Slices were then solubilized and assayed by western blotting for P-Ser 935, LRRK2 and actin level. **(B)** The graph reports P-Ser 935 level, normalized on LRRK2 level and calculated as fold over not treated sample. Data are expressed as mean ± s.e.m; ^*^p < 0.01 vs. not treated sample, *n* = 4, ANOVA followed by Dunn's *post-hoc* test.

**Figure 2:**
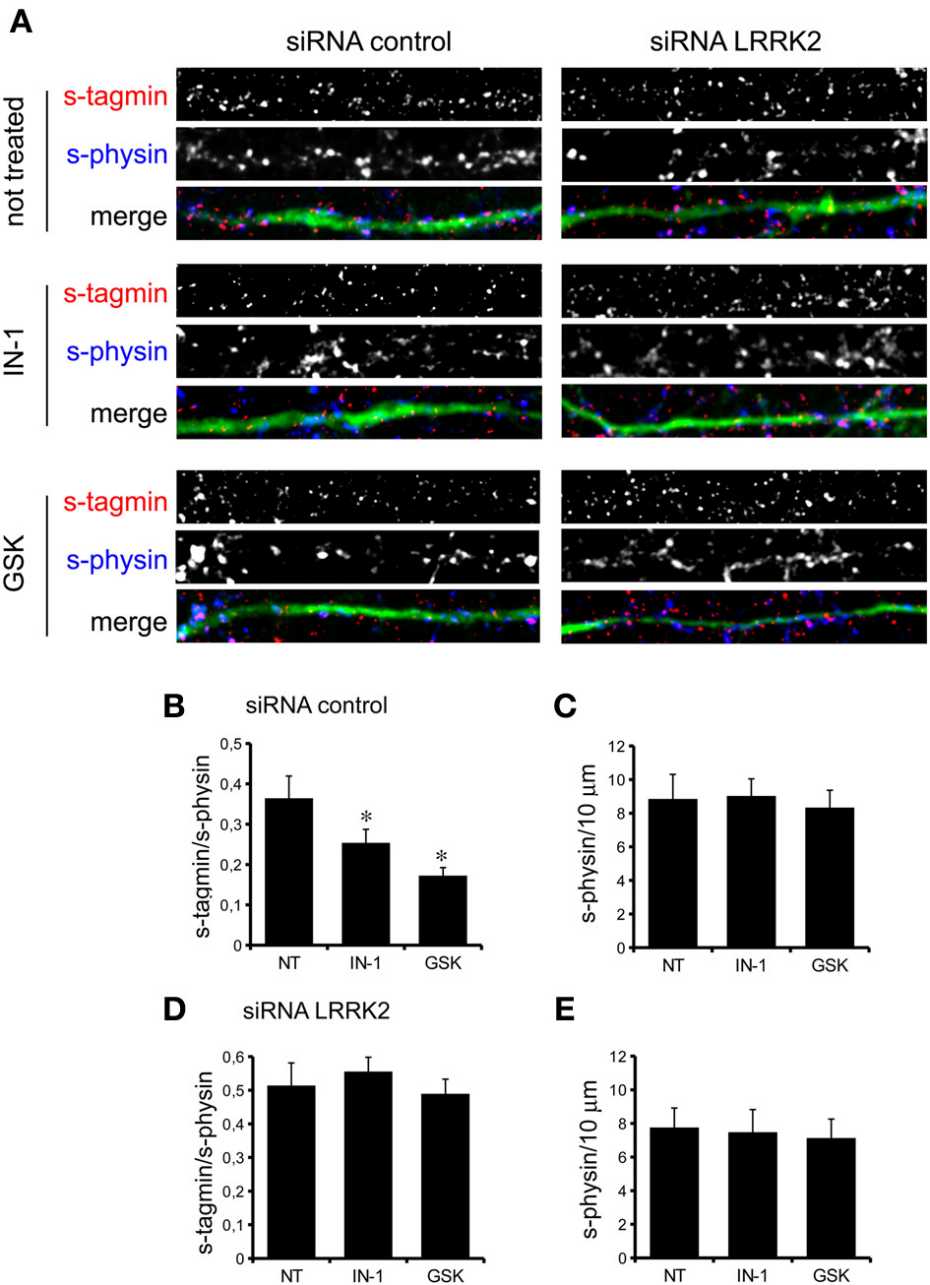
**LRRK2 kinase inhibition impairs SV trafficking. (A)** The exo-endocytotic assay was performed on cortical neurons infected at DIV4 with virus expressing control siRNA and GFP and left untreated (NT) or incubated with IN-1 or GKS compound (both 2 μ M for 2 h) before being tested at DIV14. Cycling SV appear as synaptotagmin (s-tagmin) positive clusters along neuron processes. Total SV pool was revealed by staining with anti-synaptophysin antibodies upon fixation and permeabilization. Images show signals acquired for synaptotagmin, synaptophysin and their superimposition plus GFP (merge). **(B)** The percentage of s-tagmin and s-physin positive clusters within the totality of s-physin positive clusters reflects the pool of cycling vesicles. **(C)** Total number of SV pools was not altered by treatment with IN-1 and GSK compound. The graph reports number of synaptophysin-positive clusters per 10 μm of GFP-positive process. **(D)** Similar experiments were performed on cortical neurons infected on DIV4 with viruses expressing LRRK2 siRNA and GFP. In LRRK2 down-regulated culture SV cycling is not affected upon treatment with IN-1 and GSK compound (both 2 μ M for 2 h). **(E)** Total number of SV pools was not altered by treatment with IN-1 and GSK compound. The graph reports number of synaptophysin-positive clusters per 10 μm of GFP-positive process. Data are expressed as mean ± s.e.m.; ^*^p < 0.05 vs. not treated, *n* = 20, ANOVA followed by Dunn's *post-hoc* test. Panel size is 35 × 5 μ m.

**Figure 3:**
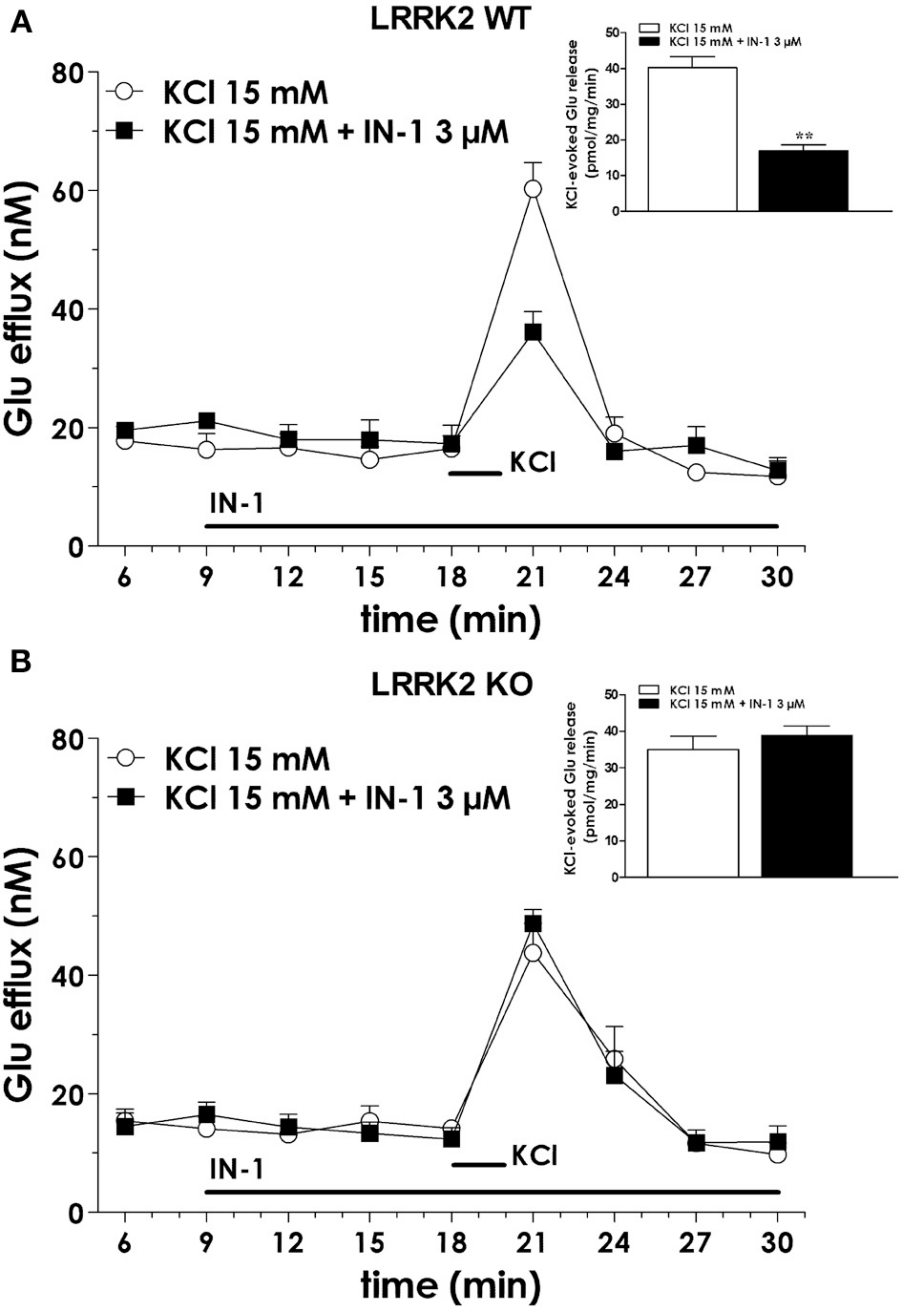
**IN-1 impairs neurotransmitter release from isolated synaptosome. (A)** Synaptosomes obtained from the cerebral (fronto-temporal) cortex of LRRK2 WT mice were perfused with Krebs solution, and stimulated with a 90 s pulse of 15 mM KCl. IN-1 (3 μ M) was perfused 9 min before KCl and maintained until the end of experiment. IN-1 reduced K^+^-evoked glutamate overflow. Data are means ± s.e.m. of 5–6 determinations per group, and are expressed as absolute glutamate concentrations in the superfusate (in nM) or K^+^-evoked glutamate overflow (in pmol/mg protein/min; insets). **(B)** Similar experiments were executed on cortical synaptosome obtained from LRRK2 KO mice. IN-1 (3 μ M) failed to impair K^+^-evoked glutamate overflow. Statistical analysis was performed on overflow values by the Student *t*-test for unpaired data. ^**^p < 0.01 different from KCl alone.

**Figure 4:**
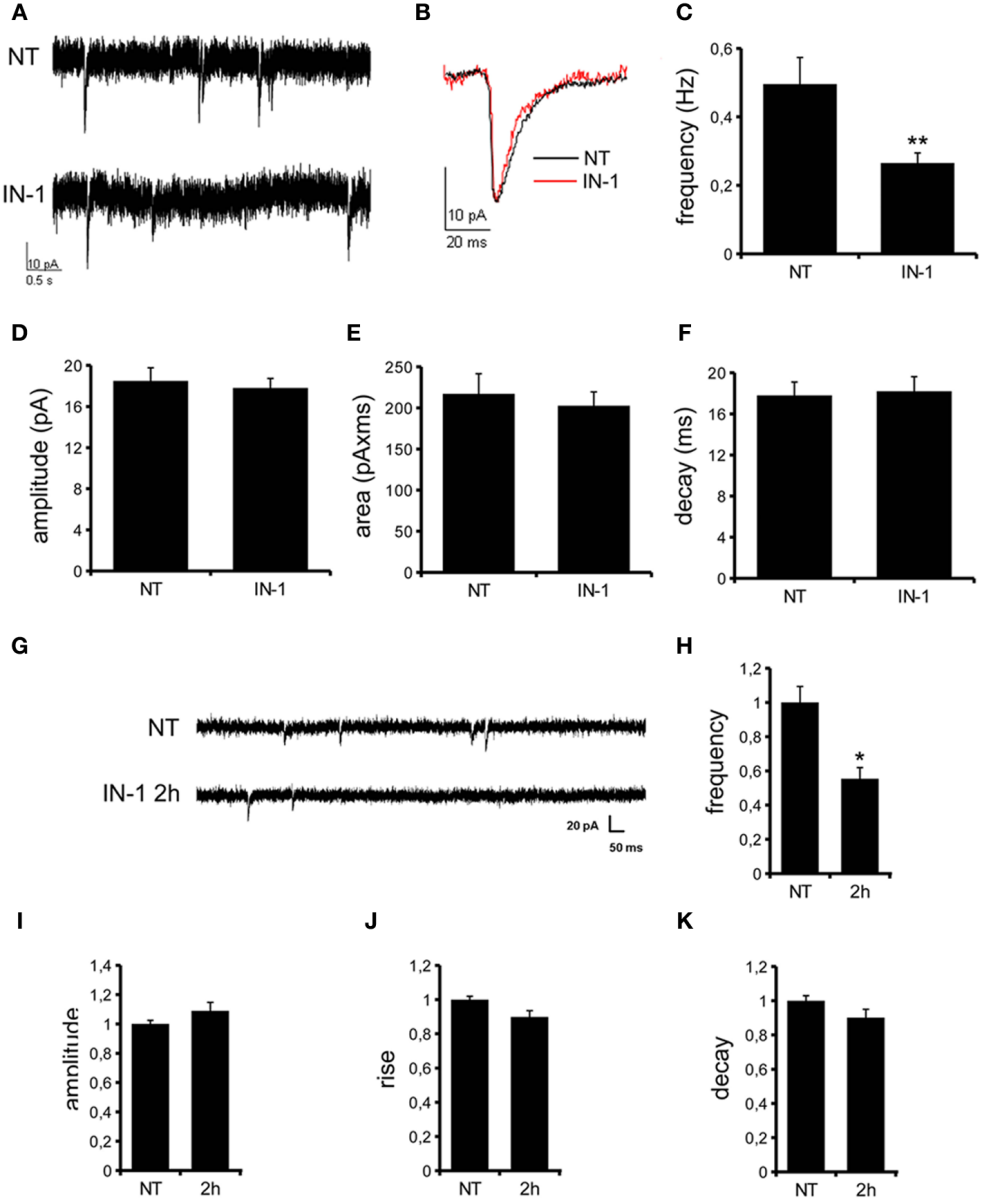
**IN-1 influences mEPSC frequency. (A)** Representative traces of mEPSCs from CA1 pyramidal neurons of hippocampal slices incubated in regular aCSF (NT) or in aCSF + IN-1 (2 μ M) for at least 2 h (IN-1). **(B)** A single mEPSC is shown. Quantification of mEPSCs basal properties reveal changes in current frequency **(C)** but not amplitude **(D)**, area **(E)** and decay **(F)**. Data are expressed as mean ± s.e.m.; ^**^p < 0.01 vs. NT, Student's *t*-test, *n* = 15. **(G)** Representative traces of mEPSCs from hippocampal culture at DIV12-13 under control condition (NT) or after incubation with IN-1 (2 μ M) for 2 h. IN-1 pretreatment reduces mEPSC frequency **(H)**, but not amplitude **(I)**, rise **(J)** or decay **(K)** time. Data were normalized on not treated condition and expressed as mean ± s.e.m.; ^*^p < 0.05 vs. not treated, *n* = 14, ANOVA followed by Dunn's *post-hoc* test.

**Figure A1:**
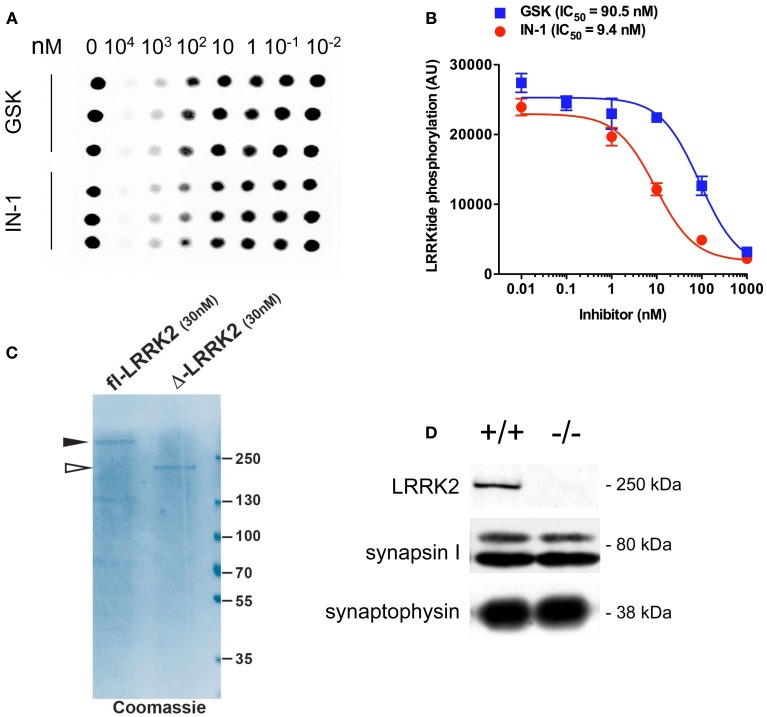
**IN-1 and GSK inhibit LRRK2 activity *in vitro*. (A)** Recombinant GST-LRRK2^970-2527^ was incubated with increasing concentrations of LRRK2 inhibitors IN-1 or GSK in the presence of 500 μM LRRKtide and 100 μM ATP (0.5 μCi ^33^P-ATP). Reactions were spotted onto P81 phosphocellulose paper and LRRKtide radioactivity quantified by phosphoimaging scanner. **(B)** Dose-response curves and calculation of IC50 values indicates that both inhibitors are active against LRRK2 but at different potencies (*n* = 6 replicates, from 2 independent set of experiments). **(C)** Full length FLAG-LRRK2 purified from transfected HEK293T cells and GST-LRRK2^970-2527^ were resolved on SDS-PAGE and visualized via coomassie staining. **(D)** Western-blotting analysis of synaptosome purified from wild-type and LRRK2 KO mice. Synapsin I level remains does not differ between the two genotypes.

We previously demonstrated that LRRK2 controls synaptic transmission acting as a presynaptic scaffold (Piccoli et al., 2011). Given that LRRK2 possesses an active kinase domain, we investigated the impact of LRRK2 kinase inhibition on synaptic activity. We modulated LRRK2 kinase activity taking advantage of two potent LRRK2 inhibitors, IN-1 (Deng et al., 2011) and GSK-2578215A (Reith et al., 2012) (hereinafter GSK). These two molecules proved to inhibit LRRK2 kinase activity when tested by *in vitro* assays where GST-LRRKtide was offered to recombinant LRRK2 (Figures A1A–C). We confirmed IN-1 and GSK efficacy on acute hippocampal slices treated with IN-1 and GSK (both 1 μM, 2 h) before solubilization and western-blotting. LRRK2 kinase activity, indirectly monitored by P-Ser 935 level, was clearly impaired upon incubation of slices with IN-1 and GSK (Figures 1A,B). Thus, we monitored the effect of kinase inhibition on presynaptic SV recycling by exposing living culture to rabbit polyclonal antibodies directed against the intravesicular domain of synaptotagmin1, which are internalized inside the vesicle lumen upon SV recycling (Matteoli et al., 1992). Cortical cultures were infected at DIV4 with control viruses co-expressing GFP to track neuronal processes and assayed at DIV14. Prior to these assays, primary cultures were treated with vehicle (DMSO) or IN-1 or GSK (both 2 μM for 2 h). Vesicles within GFP positive processes were then monitored via laser confocal microscopy. The vesicles appeared as clusters either synaptotagmin and synaptophysin positive (i.e., cycling vesicles) or only synaptophysin positive (Figure 2A). The analysis showed that LRRK2 inhibition via either IN-1 or GSK induced a significant decrease in the number of synaptotagmin and synaptophysin positive clusters (Figure 2B). The total number of synaptic contacts, however, remained unaltered despite any pharmacological treatments (Figure 2C). A number of off-targets has been described for LRRK2 IN-1, including ERK5 (Luerman et al., 2014). Thus, in order to determine if the effect reported was specifically related to LRRK2 kinase inhibition, we quantified SV recycling rate in cultures infected at DIV4 with viruses expressing LRRK2 silencing constructs, siRNA LRRK2 (Bauer et al., 2009; Piccoli et al., 2011). As previously reported, LRRK2 silencing was associated to an increase in synaptotagmin uptake (Piccoli et al., 2011). Interestingly, we did not measure any significant alteration of SV recycling rate or number of synaptic contact upon IN-1 or GSK treatment in LRRK2 silenced culture (Figures 2D,E). These results likely exclude that the effect seen on SV trafficking arises from substantial off target effect of IN-1 or GSK. Furthermore, our data suggest that IN-1 and GSK have similar efficacy in terms of kinase inhibition and impact on SV trafficking. Based on the equivalent behavior of the two inhibitors, in the subsequent functional assays we focused mainly on IN-1. Given the impact of LRRK2 inhibition on SV trafficking, we next investigated the effect on IN-1 on neurotransmitter release. To this aim we measured glutamate release from isolated synaptosomes upon IN-1 treatment in either basal or stimulated condition (Figure 3A). A pulse of 15 mM K^+^ caused an approximate three-fold, transient elevation of glutamate levels. IN-1 (3 μM) did not affect spontaneous glutamate efflux, but inhibited the K^+^-evoked glutamate overflow by about 60%. In a complementary approach, we measured the basal and evoked (15 mM K^+^) release of [3H]D-aspartate in presence or not of IN-1 (1 μM). Also in this model, IN-1 impaired the K^+^ evoked release by about 35% (calculated as fraction of overflow and expressed as mean ± s.e.m.: K^+^ alone = 1.4 ± 0.01 K^+^ + IN-1 = 0.9 ± 0.01, *p* < 0.01, *n* = 7, Student's *t*-test). To exclude potential off target effect of IN-1, we studied glutamate release in synaptosomes obtained from LRRK2 KO mice (Figure 3B). Spontaneous and K^+^-evoked glutamate efflux was not different between the two genotypes. However, IN-1 (3 μM) did not significantly influence the K^+^-evoked glutamate release in LRRK2 KO mice. Robust evidence correlates synapsin I to the mobilization of SV and release of neurotransmitter (Orenbuch et al., 2012). Thus, we verified whether the lack of effect of IN-1 on glutamate release we reported in LRRK2 KO mice could arise from disturbed synapsin I level. Western blotting analysis of synaptosome from wild-type and LRRK2 KO mice did not shown any significant difference (Figure A1D). This evidence indicates that the impairment in neurotransmitter release arises from a specific effect of IN-1 on LRRK2. Given the impact of LRRK2 kinase inhibition on presynaptic functions, we next evaluated the functional outcome of LRRK2 inhibition in terms of neuronal activity. To this aim, we studied the electrophysiological properties in two different neuronal models, namely acute hippocampal slices and hippocampal cultures. First we exposed acute hippocampal slices obtained from wild-type mice at P21-22 to IN-1 2 μM for 2 h before electrophysiological recording in CA1 region. We detected a clear reduction in miniature excitatory responses (mEPSCs) frequency in the absence of any change in amplitude, rise, or decay time upon acute IN-1 treatment (Figures 4A–F). In order to validate the impact of LRRK2 inhibition, we measured the electrophysiological properties of primary hippocampal neurons treated with IN-1, 2 μM for 2 h. Our experiments demonstrated that acute IN-1 treatment reduces mEPSC frequency but not amplitude, rise or decay time (Figures 4G–K). Similar results were obtained by treating cells with GSK (1 μM, 2 h; *NT* = 1 ± 0.21, GSK = 0.5 ± 0.09, *p* = 0.057, *n* = 6; values represent frequency normalized on untreated cultures and expressed as mean ± s.e.m.). These experiments indicate that the pharmacological inhibition of LRRK2 kinase activity reduces synaptic transmission affecting SV recycling and thus neurotransmitter release.

### Kinase activity controls LRRK2 binding properties

**Figure 5:**
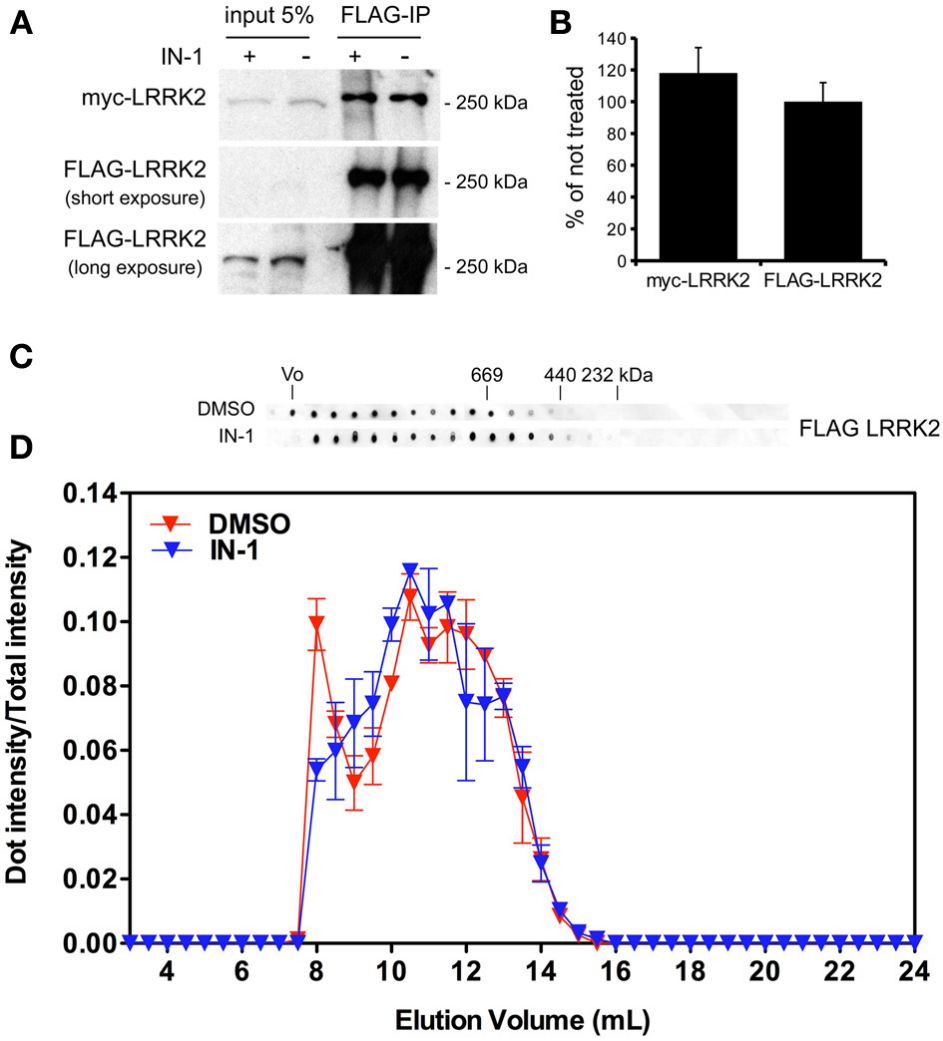
**IN-1 does not influence LRRK2 dimerization. (A)** HEK293T cells expressing both myc and FLAG LRRK2 were treated or not with IN-1 (2 μ M for 2 h), solubilized and processed for FLAG immunopurification. We evaluated the extent of LRRK2 homodimerization by measuring the amount of myc LRRK2 co-precipitating with FLAG LRRK2 **(B)** The graph reports the amount of FLAG and myc LRRK2 recovered in FLAG immunoprecipitates upon IN-1 incubation. Data were calculated as fraction of untreated sample and expressed as mean ± s.e.m. (*n* = 4). **(C)** Full-length LRRK2 purified by Flag immunoaffinity from untreated HEK293T cells was separated by size exclusion chromatography (SEC) and subsequently treated or not with IN-1 (1 μ M, 90 min on ice). **(D)** The intensity of each dot (fraction) is normalized by the integrated intensities. Column void volume is 7.5 ml.

**Figure 6:**
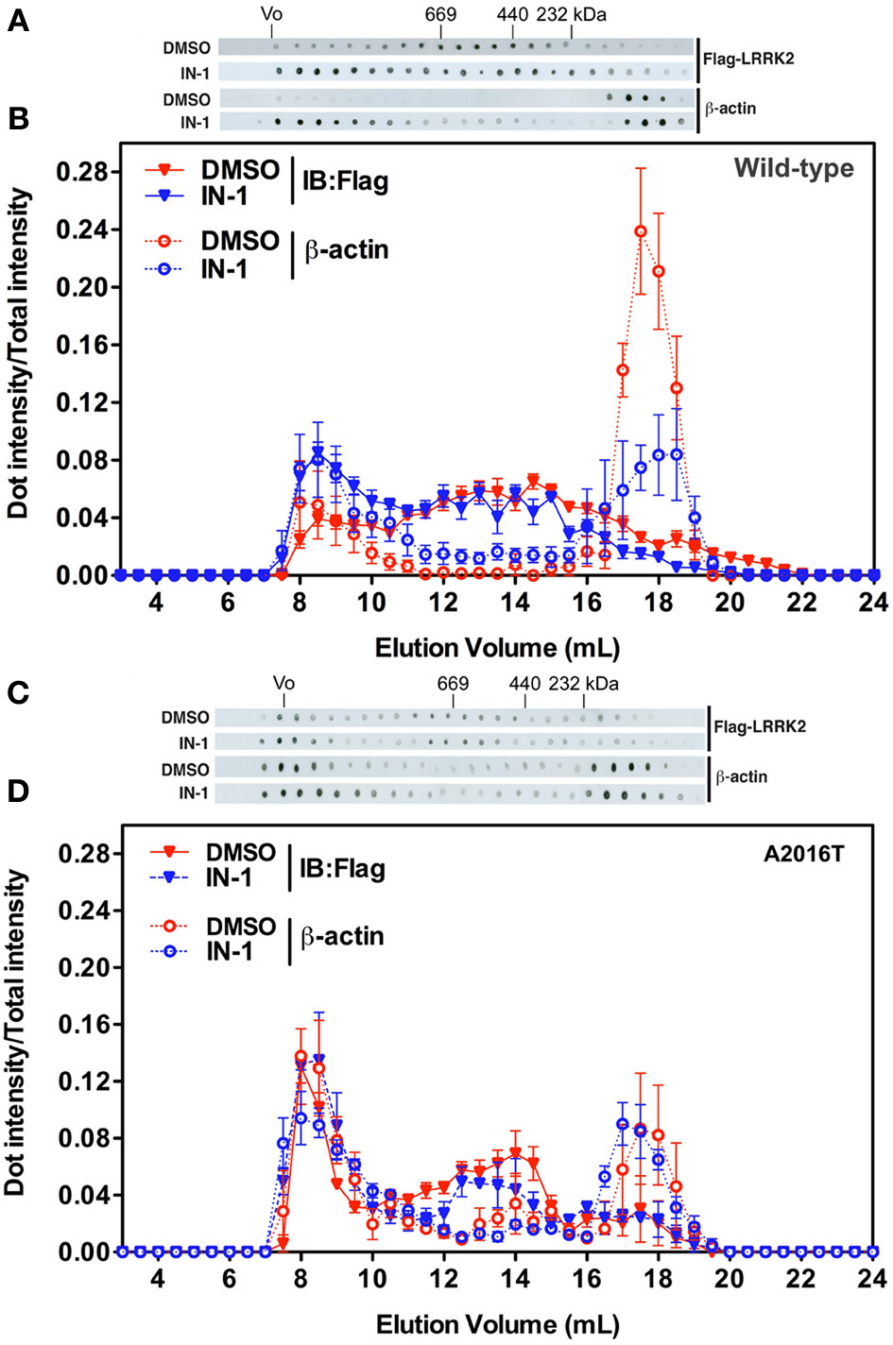
**IN-1 alters LRRK2 macromolecular complex. (A)** HEK293T cells expressing FLAG-LRRK2 wild-type were treated or not with IN-1 (1 μ M, 90 min), solubilized and then separated by SEC. FLAG-LRRK2 and actin were revealed by western-blotting of fractions spotted on nitrocellulose. **(B)** The intensity of each dot (fraction) is normalized by the integrated intensities. **(C)** HEK293T cells expressing FLAG-LRRK2 A2016T were treated or not with IN-1 (1 μ M, 90 min), solubilized and then separated by SEC. LRRK2 and actin were revealed by western-blotting of fractions spotted on nitrocellulose. **(D)** The intensity of each dot (fraction) is normalized by the integrated intensities.

**Figure 7:**
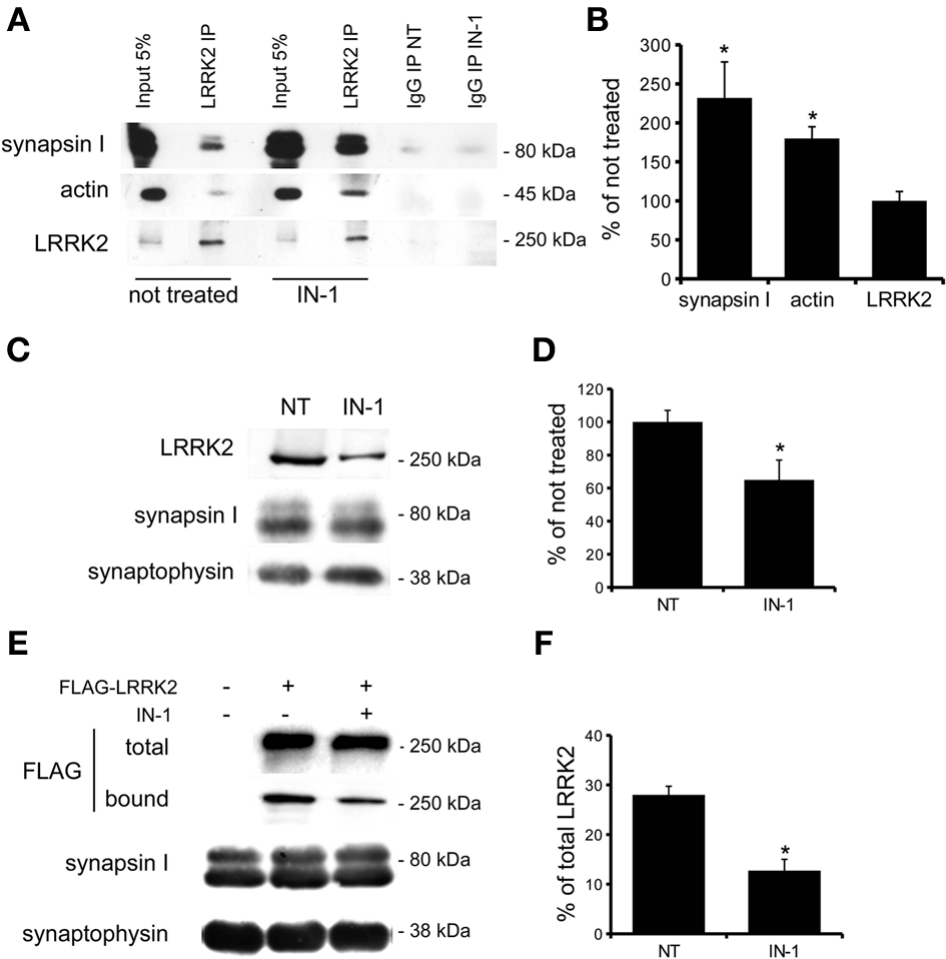
**IN-1 modifies LRRK2 binding properties. (A)** Extracts of purified cortical synaptosomes were incubated with anti-LRRK2 antibodies or rabbit IgG in absence (not treated) or in presence of IN-1 (IN-1, 1 μ M 2 h). The immunocomplexes were sedimented with protein G-Sepharose and the samples were resolved by SDS-PAGE and analyzed by immunoblotting with anti synapsin I, anti actin and anti LRRK2 antibodies. **(B)** Quantification of IN-1 effect on LRRK2 interaction with synapsin I, actin and LRRK2 itself. Results are calculated as percent of respective controls (not treated sample) and expressed as mean ± s.e.m. (^*^P < 0.05, *n* = 4, Student's *t*-test vs. not treated sample). **(C)** purified native synaptic vesicles (SV; 40 μ g/sample) were incubated in the absence (NT) or presence of IN-1 (IN-1, 1 μ M 2 h). After incubation, SV were recovered by high speed centrifugation and the residual amounts of endogenous LRRK2 bound to SV were determined by immunoblotting with anti-LRRK2 antibodies. The recovery of SV in the pellet was evaluated based on synaptophysin immunoreactivity. **(D)** LRRK2 recovery in the SV pellet was calculated as the percentage of the not treated sample and shown as mean ± s.e.m. (^*^P < 0.05, *n* = 8, Student's *t*-test vs. relative control). **(E)** Purified FLAG-LRRK2 was incubated with SV (10 μ g protein/sample) in presence or absence (NT) of IN-1 (IN-1, 1 μ M 2 h). SV-bound FLAG-LRRK2 was separated from free FLAG-LRRK2 by high-speed centrifugation and quantified by immunoblotting with anti-FLAG antibody. The recovery of SV in the pellet was evaluated based on synaptophysin immunoreactivity. **(F)** The binding of FLAG-LRRK2 to SV was calculated as the percentage of total FLAG-LRRK2 and expressed as mean ± s.e.m. ^*^p < 0.05; Student's *t*-test vs. relative control.

Independent studies demonstrated that LRRK2 exists in multiple oligomeric state: kinase-active dimer (Deng et al., 2008; Greggio et al., 2008; Klein et al., 2009) and monomers or oligomers mainly inactive (Sen et al., 2009). Thus, we asked whether LRRK2 kinase inhibition might influence LRRK2 oligomeric state. First we explored whether kinase inhibition affects LRRK2 homologous interaction by evaluating the extent of LRRK2 dimerization in presence of IN-1. To this aim we co-expressed FLAG-LRRK2 and myc-LRRK2 in HEK293T cells; we subsequently treated the cell with IN-1 (2 h, 1 μM) and eventually we immobilized LRRK2 on FLAG-M2 beads. After elution, we measured the recovery of FLAG and myc LRRK2 by immunoblotting with specific anti tag antibodies (Figure 5A). We found that IN-1 treatment does not significantly affect the amount of myc LRRK2 co-precipitating with FLAG-LRRK2 (Figure 5B). To further explore the impact of kinase inhibition on LRRK2 oligomerization, we performed size exclusion chromatography (SEC) experiments on FLAG-LRRK2 proteins purified from untreated cells and then incubated with IN-1 (1 μM, 90 min). As shown in Figures 5C,D, the elution profile of purified LRRK2 is only marginally affected by IN-1 inhibition. We obtained comparable results incubating FLAG-LRRK2 with GSK (1 μM, 90 min, data not shown). These data suggest that kinase inhibition minimally impacts LRRK2 oligomeric state. Next, we asked whether kinase inhibition engages LRRK2 in differential heterologous interactions. We have previously demonstrated that LRRK2 interacts with a panel of proteins, including actin (Piccoli et al., 2011). Thus, we analyzed by SEC the elution profile of FLAG-LRRK2 and actin in lysates extracted from cells treated with IN-1 (1 μM, 90 min). Interestingly, we observed that IN-1 shifted both LRRK2 and actin toward higher molecular weight forms and that the two elution profiles partially overlap (Figures 6A,B). This outcome might be consistent with the possibility that LRRK2 forms higher molecular weight complexes with actin upon IN-1 binding. To further substantiate this hypothesis we over-expressed FLAG-LRRK2 A2016T, an artificial variant unable to bind IN-1, in HEK293T cells (Nichols et al., 2009; Deng et al., 2011). When we analyzed by SEC the elution profile of FLAG-LRRK2 A2016T and actin in lysates extracted from cells treated with IN-1 (2 h, 1 μM), we observed that IN-1 failed to shift either LRRK2 or actin elution profiles (Figures 6C,D). All together these data strongly suggest that kinase inhibition induces the formation of high-molecular weight complexes including LRRK2 and its interacting partners. To further explore this hypothesis, we asked whether LRRK2 inhibition might affect LRRK2 affinity toward SV associated proteins such as synapsin I and actin. To this aim we immunoprecipitated LRRK2 with anti-LRRK2 antibodies [MJFF C41-2] using purified synaptosomes treated with IN-1 (1 μM) during the assay as protein source. We found that the binding of LRRK2 to synapsin I and actin increased in presence of IN-1 (Figures 7A,B). Given the effects of kinase inhibition on LRRK2 binding features, we first investigated if LRRK2 binds to SV and, next, if kinase inhibition disturbs LRRK2 and synapsin I binding to SV. To this aim we incubated native purified SV (in the range of 40 μg/sample) under phosphorylation permissive conditions or in the presence of IN-1 (1 μM, 1 h). After incubation, we recovered SV by high speed centrifugation and determined the amounts of bound LRRK2 and synapsin I by immunoblotting. The SV recovery in the pellet was evaluated based on synaptophysin immunoreactivity. We found that LRRK2 binds SV and that this interaction is significantly decreased in the presence of IN-1 while synapsin I binding to SV was unaffected by IN-1 (Figures 7C,D). As a complementary approach we analyzed the impact of IN-1 (1 μM, 1 h) on the interaction between SV and exogenous recombinant FLAG-LRRK2 (Figure A1C). After incubation, we separated SV-bound LRRK2 by high-speed centrifugation and evaluated the recovery of SV and bound LRRK2 and synapsin I in the pellet by immunoblotting. Our data showed that IN-1 significantly reduces exogenous LRRK2 binding to SV while the yield of SV-bound synapsin I remains unaltered (Figures 7E,F). This evidence suggests that kinase inhibition interferes with the macro-molecular complex bound to LRRK2 at the presynaptic site.

## Discussion

**Figure 8:**
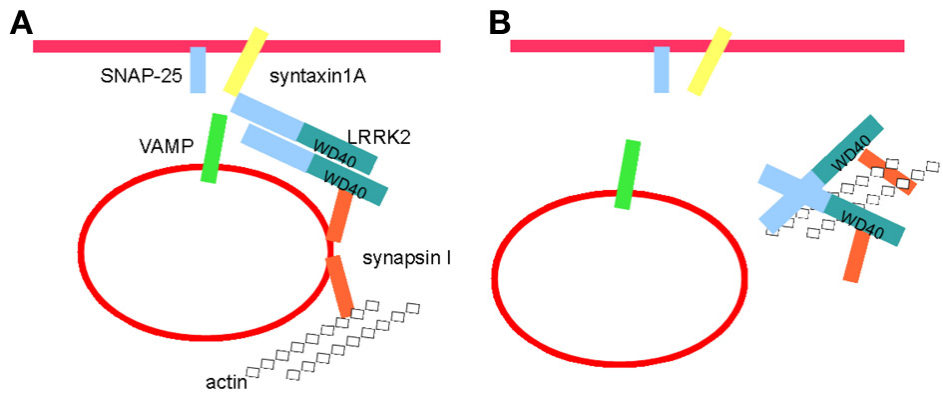
**Kinase activity controls LRRK2 molecular complex at the synaptic site. (A)** LRRK2 binds SV and regulates thier trafficking via interaction with a panel of presynaptic proteins, including synapsin I, syntaxin 1, SNAP-25, VAMP, and actin (see also Piccoli et al., 2011, 2014). **(B)** Kinase inhibition detaches LRRK2 from SV and might induce the formation of high-molecular weight complex including LRRK2, synapsin I and actin. Such complex might sequester LRRK2, synapsin I and actin thus hampering their function within SV cycle.

Our previous observations provided evidence that LRRK2 executes critical functions at the presynaptic site; given its relative position as an integral part of a presynaptic protein network, LRRK2 may serve as a molecular hub coordinating both the storage and the mobilization of SV driven by activity (Piccoli et al., 2011, 2014). Recent work has clarified that LRRK2 controls SV in the ready releasable pool via inhibitory phosphorylation of the SNAP-25 interacting protein Snapin (Yun et al., 2013). The evidence reported here adds one more level of complexity: the implication of LRRK2 kinase activity within synaptic functions. As wild type LRRK2 is characterized by a low kinase activity (MacLeod et al., 2006), it might be argued that physiologically LRRK2 acts as a scaffold protein and its kinase activity mainly regulates its macro-molecular organization. In fact, several independent studies have revealed that LRRK2 exists in different forms in equilibrium, namely monomer, dimer and oligomer, being the dimer the predominant status under native conditions (Sen et al., 2009; but see Ito and Iwatsubo, 2012). Interestingly, PD associated LRRK2 mutations disturb both LRRK2 dimerization (Sen et al., 2009) and ternary complex formation (Nichols et al., 2010). Furthermore, acute treatment with IN-1 induces the aggregation of ectopic LRRK2 expressed in heterologous cell lines and interferes with 14-3-3 binding (Deng et al., 2011). Our hypothesis is that kinase inhibition triggers the formation of high molecular weight complexes encompassing LRRK2 and LRRK2 interacting proteins. These phenomena might affect LRRK2 function at the presynaptic site. Accordingly, we described that kinase inhibition reduces LRRK2 binding to SV and at the same time increases LRRK2 affinity toward actin and synapsin I. As suggested by the SEC analysis of LRRK2 expression in heterologous lines, we can speculate that kinase inhibition induces the formation of LRRK2 high molecular weight complex also within neuronal cells. Such complex might act as dominant-negative on synaptic function. In fact it might not only sequester LRRK2 but also free actin and synapsin I, making them unavailable for physiological binding to SV (Figure 8). Indeed we did not report a clear reduction of synapsin I bound to SV upon IN-1 incubation or in presence of exogenous FLAG-LRRK2. This lack of an effect could arise from the design of our experimental setup. In fact we treated with IN-1 and/or FLAG-LRRK2 purified SV isolated from their cellular context and incubated in artificial buffer. LRRK2, synapsin I and actin regulate SV mobility from intra-bouton pool to membrane where eventually SV fuse (Greengard et al., 1993; Piccoli et al., 2011; Orenbuch et al., 2012). Given the pivotal role of these three proteins during SV cycle and the biochemical consequences of kinase inhibition on the stability of LRRK2 binding with actin and synapsin I on one side and with SV on the other, not surprisingly kinase inhibition impairs proper SV cycling, reduces neurotransmitter release and, eventually, decreases synaptic activity. It remains unclear how kinase activity can interfere with LRRK2 biochemical features. In particular, kinase inhibition might modulate LRRK2: 1) by reducing cis or trans homo-phosphorylation thus affecting homodimerization and/or 2) by impairing binding of LRRK2 interacting proteins and/or 3) by abolishing phosphorylation of LRRK2 substrates themselves involved in regulating LRRK2 macro-molecular complex. While our studies do not address the third hypothesis, our present work favors the possibility that kinase activity drives the organization of LRRK2 macro-molecular complex. In fact, in our hands LRRK2 kinase inhibition does not have a major effect on LRRK2 dimerization but instead increases affinity toward interacting partners. Furthermore, while we executed a number of experiments in intact cells where potential LRRK2 interactors as well as signaling machinery up or down stream to LRRK2 kinase activity might be present and functional, we addressed the effect of kinase inhibitors also on isolated protein, i.e., purified LRRK2 extrapolated from its cellular context. In particular, we reported that upon IN-1 incubation purified LRRK2 binds less efficiently SV. Thus, a fourth intriguing hypothesis is that the binding itself of IN-1 affects LRRK2 properties. A recent observation suggested that IN-1 binding into the activation segment within kinase domain induces a wide-spread conformational change (Gillardon et al., 2013). Thus, it is possible that the structural stress consequent to IN-1 binding slightly modifies LRRK2 folding and thus impairs LRRK2 binding to SV. Given that our SEC analysis of isolated LRRK2 failed to reveal any significant effect of IN-1, we predict such effect to be minor or at least not able to robustly perturb LRRK2 oligomerization. Although further studies are indeed required to fully dissect these different hypothesis, overall our data indicate that LRRK2 function at the synaptic site depends on kinase regulation of LRRK2 macro-molecular organization. This might play a critical role also in PD pathogenesis. In fact severe synaptic defects have been reported in different models expressing kinase hyper-active LRRK2 (reviewed in Belluzzi et al., 2012). In particular, G2019S transgenic mice display an altered striatal DA release (Li et al., 2010; Melrose et al., 2010) and the overexpression of mutant G2019S influences SV trafficking rates (Shin et al., 2008; Yun et al., 2013). Thus, synaptic activity might arise as a key pathway affected by LRRK2 mutation. Being G2019S predicted as a gain-of-function mutation, huge effort has been spent to develop specific LRRK2 inhibitors. However, the side effect on kidney described upon chronic treatment with LRRK2 inhibitor (Herzig et al., 2011) together with the functional implication of LRRK2 endogenous kinase activity here described, suggest that other therapeutic strategies might result necessary. Our work suggests that the regulation of LRRK2 complex is a crucial molecular actor implicated in LRRK2 physiological function and demonstrate the necessity to tackle LRRK2 biology beyond its kinase activity.

## Author contributions

Maria D. Cirnaru, Antonella Marte, Elisa Belluzzi, Isabella Russo, Martina Gabrielli, Francesco Longo, Ludovico Arcuri, Luca Murru performed experiments; Luigi Bubacco, Michela Matteoli, Ernesto Fedele, Carlo Sala, Maria Passafaro, Michele Morari, Elisa Greggio, Franco Onofri, and Giovanni Piccoli analyzed data; Michele Morari, Elisa Greggio, Franco Onofri, and Giovanni Piccoli designed experiments and wrote the paper.

### Conflict of interest statement

The authors declare that the research was conducted in the absence of any commercial or financial relationships that could be construed as a potential conflict of interest.
